# Rate, Risk Factors, and Causes of Neonatal Deaths in Jordan: Analysis of Data From Jordan Stillbirth and Neonatal Surveillance System (JSANDS)

**DOI:** 10.3389/fpubh.2020.595379

**Published:** 2020-10-30

**Authors:** Nihaya A. Al-Sheyab, Yousef S. Khader, Khulood K. Shattnawi, Mohammad S. Alyahya, Anwar Batieha

**Affiliations:** ^1^Faculty of Applied Medical Sciences, Jordan University of Science and Technology, Irbid, Jordan; ^2^Department of Public Health and Community Medicine, Faculty of Medicine, Jordan University of Science and Technology, Irbid, Jordan; ^3^Department of Maternal and Child Health Nursing, Faculty of Nursing, Jordan University of Science and Technology, Irbid, Jordan; ^4^Department of Health Management and Policy, Faculty of Medicine, Jordan University of Science and Technology, Irbid, Jordan

**Keywords:** neonate, mortality, risk factor, surveillance, Jordan

## Abstract

**Background:** It has been estimated that 27.8 million neonates will die worldwide between 2018 and 2030 if no improvements in neonatal and maternal care take place. The aim of this study was to determine the rate, risk factors, and causes of neonatal mortality in Jordan.

**Methods:** In August 2019, an electronic stillbirths and neonatal deaths surveillance system (JSANDS) was established in in three large cities through five hospitals. Data on all births, neonatal mortality and their causes, and other characteristics in the period between August 2019 and January 2020 were exported from the JSANDS and analyzed.

**Results:** A total of 10,328 births [10,226 live births (LB) and 102 stillbirths] were registered in the study period, with a rate of 14.1 deaths per 1,000 LBs; 76% were early neonatal deaths and 24% were late deaths. The odds of deaths in the Ministry of Health hospitals were almost 21 times (OR = 20.8, 95% CI: 2.8, 153.1) higher than that in private hospitals. Low birthweight and pre-term babies were significantly more likely to die during the neonatal period compared to full-term babies. The odds of neonatal mortality were significantly higher among babies born to housewives compared to those who were born to employed women (OR = 2.7; 95% CI: 1.2, 6.0). Main causes of neonatal deaths that occurred pre-discharge were respiratory and cardiovascular disorders (43%) and low birthweight and pre-term (33%). The main maternal conditions that attributed to these deaths were complications of the placenta and cord, complications of pregnancy, and medical and surgical conditions. The main cause of neonatal deaths that occurred post-discharge were low birthweight and pre-term (42%).

**Conclusions:** The rate of neonatal mortality have not decreased since 2012 and the majority of neonatal deaths occurred could have been prevented. Regular antenatal visits, in which any possible diseases or complications of pregnant women or fetal anomalies, need to be fully documented and monitored with appropriate and timely medical intervention to minimize such deaths.

## Background

Neonatal mortality is a public health problem worldwide primarily in low- and middle-income countries. Although extensive progress has been completed in reducing neonatal mortality over the last three decades, increased efforts to improve progress are still needed to achieve the 2030 SDG target (1). Even though there is a global decrease in neonatal mortality, the rate of decrement is considerably lower than that of the post-neonatal under five mortalities (2).

It has been estimated that 27.8 million neonates will die between 2018 and 2030 (1) if no improvements in neonatal mortality take place. A study conducted in 186 countries revealed that the risk of early neonatal death is very high across a range of countries and contexts (3). Of all neonatal deaths, about half occurred within 24 h of birth and around one third occurred in the first 6 h after birth (4). According to the Jordan Perinatal and Neonatal Mortality study (5), the neonatal mortality rates was 14.9 per 1,000 live births (LB).

In low- and middle-income countries, the majority of neonatal deaths occur without a clear cause of death (i.e., pre-maturity) (6, 7). It is difficult to confirm the cause because there are many factors that could be linked to the exact underlying cause of neonatal mortality, however, literature has categorized causes into those related to maternal or fetal conditions (8). Neonatal deaths often occur due to an illness presenting as an emergency, either soon after birth or later, due to infections such as tetanus or community-acquired infections (9). Data on causes of neonatal deaths and the timing around neonatal deaths are often sparse and less reliable than all-cause mortality data, and these data result in uncertain estimates, which poses substantial challenges to the generation of evidence-based interventions to prevent neonatal deaths. Improved data on where and when neonatal death occur and what causes delays is key to designing context-specific community and strategies (9).

The scarcity of data in Jordan on stillbirths and neonatal mortality, especially early mortality is generally linked to the fact that some births are not registered (10, 11). In addition, the existing sources of data on neonatal mortality are likely to be biased or incomprehensive. Given the fact that almost all (99.7%) births occur in Jordan are institutionalized (the birth occurs in hospital) (12), improving a reporting system of neonatal deaths in Jordanian hospitals is critical for tracking progress and taking appropriate actions.

As a result of this limitation, an electronic stillbirths and neonatal deaths surveillance system (JSANDS) was developed and established in five large hospitals in Jordan in August 2019. The JSANDS was developed by researchers at a leading university in Jordan with the collaboration of the Jordanian Ministry of Health as a secure on-line data entry system to collect, organize, analyze, and disseminate reliable data on neonatal deaths, and related causes. Additionally, the system registers births to use them as a denominator for mortality measures (13). The definition of the stillbirths and neonatal deaths used in the system were based on the international standards set by the World Health Organization and CDC. Given the scarcity, incomprehensiveness, and inconsistency of national data about causes and risk factors of stillbirths and neonatal mortality, the current study utilized the data from JSANDS aiming to determine the rate, risk factors, and causes of neonatal mortality in Jordan.

## Methods

### Study Population

The information on all births and related outcomes that registered in the JSANDS surveillance system from August 2019 to January 2020 were retrieved and analyzed. We included all the births and deaths (stillbirths and neonates) within the indicated 6-months period of data collection. Any birth, stillbirth, or a neonatal death occurred within the five hospitals and entered into the JSANDS system were included in the study. If the birth or death is not registered on the JSANDS, they will be excluded.

The authors understand that the study period is limited and not long enough. However, within this 6 months' period, there were a total of over than 10,000 registered births on the JSANDS that give us a glimpse of the NMR and related causes in Jordan. It is worth mentioning that the total births in Jordan during 2019 are 197,278 in 2019 (14). Based on this number, the half yearly total births in 2019 is 98,650. Thus, the % of coverage by the five included hospitals out of the total births in Jordan during 2019 is 9.55%. We will utilize the data registered in the JSANDS to run periodic, perhaps annual, analysis of the rate, causes, and risk factors of Neonatal mortality rate in Jordan.

All births and neonatal deaths occurred in the five selected hospitals were completely registered. These five selected hospitals, from three major governorates, cover the vast majority of births and deaths occur in all regions in the north, east, and south of Jordan. In particular, we included one university teaching, referral hospital in Northern Jordan, which receives labor and delivery cases from all regions and suburbs in Northern Jordan. We also included another university teaching, governmental hospital affiliated with the Ministry of Health in Northern Jordan, which is specialized for maternal-related issues including births, and also receives maternal deliveries from all sectors in the region. A big private hospital in the North of Jordan was also included from several private and governmental sectors in the region. In the north east region, we included a large specialized hospital for maternal and child health and covers the whole Al-Mafraq governorate. Finally, we selected another specialized, referral hospital in the Southern region of Jordan which covers the majority of births within the region.

The extracted data included sociodemographic characteristics of the mother and father, birth data (i.e., gestational age, mode of delivery, and multiplicity), the newborn data (status, birth weight) and causes of neonatal deaths. In the current study, neonatal mortality was defined as any death that happened within the first 28 days of life. Neonatal mortality rate was calculated as the number of neonatal deaths per 1,000 live births (LB). The definition of the stillbirths and neonatal deaths used in the system were based on the international standards set by the World Health Organization and Centre for Disease Control.

Causes of neonatal deaths were identified according to the International Classification of Diseases-Perinatal Mortality (ICD-PM), which is part of the 10th version of the International Classification of Diseases (ICD-10) and report perinatal deaths (15). A training was held in the five hospitals for all healthcare providers on how to assign cause of death. The doctor (usually pediatrician) who is responsible for the follow up of the neonate has the primary responsibility to fill the form for the death, assign the cause of death, and write the ICD-10 code accordingly. The ICD-10 codes were used to provide a unified language for reporting and monitoring diseases allowing a standardized comparison and sharing of data among the five hospitals.

Causes of deaths related to fetal/neonatal condition or related to maternal condition were registered. First, the main disease or condition in the newborn is identified, in which the main disease or condition of the newborn who has died is entered. Other diseases or conditions in the newborn were also reported, if any. The main underlying maternal disease or condition affecting the newborn that contributed mostly to the neonatal death was then reported.

Last but not least, the research team verified all deaths registered through the JSANDS with those documented on paper and electronic medical records in the hospital to avoid any missing death. We found only 1% inconsistency between deaths registered through the JSANDS and those registered on paper mainly due to some delay in entering the death case to the JSANDS. However, all deaths tend to be registered on the JSANDS within 1 day of the occurrence of death.

### Data Analysis

The IBM SPSS version 24 (IBM Corp. Released 2016. IBM SPSS Statistics for Windows, Version 24.0. Armonk, NY: IBM Corp) was used for data analysis. Data were described using rates and percentages for categorical data and means and standard deviations for continuous variables. The NMR was calculated as the number of neonatal deaths by 1,000 LB. The distribution of neonatal deaths according to studied characteristics were tested using Chi-square test. Multivariable analysis using binary logistic regression was used to determine factors associated with neonatal mortality. Backward stepwise selection method was used to select the variables to be included in the regression model. A *p*-value of <0.05 was considered statistically significant.

## Results

### Women's Demographic and Maternal Characteristics

During the period from August 2019 to January 2020, a total of 10,328 births (10,226 LB and 102 stillbirths) were registered. The women's age ranged between 15 and 48 years with a mean (SD) of 29.1 (6.1) year. The majority of women (81%) were between 19 and 35 years of age, and 16.5% were older than 35 years. More than half of women (55%) had high school education or less. Almost three quarters (74%) of women had family income <714 US$. The majority of women were housewives (90%). The rate of cesarean section rate was 49% (27% planned CS and 22% emergency CS) (Table 1).

**Table 1:** Neonatal mortality rate according to the sociodemographic, maternal, clinical and relevant characteristics of women and births' characteristics.

**Variables**	**Live births (*n*)**	**Number of deaths**	**Neonatal death rate per 1,000 live births**	***p*-value**
**Hospital**				<0.001
Private	1,795	1	0.6	
Teaching	1,432	16	11.2	
Ministry of Health	6,999	127	18.1	
**Mother age (year)**				0.737
≤ 18	253	3	11.9	
19–35	8,288	114	13.8	
>35	1,685	27	16.0	
**Mother education level**				0.028
High school or less	5,643	92	16.3	
Diploma	709	9	12.7	
Bachelor	2,476	20	8.1	
Master or higher	259	2	7.7	
Unknown	1,139	21	18.4	
**Total monthly income (US$)**				0.119
<714	7,558	112	14.8	
714–<1,428	1,699	15	8.8	
≥1,428	215	2	9.3	
Unknown	754	15	19.9	
**Working status**				0.015
Housewife	9,174	138	15.0	
Employed	1,052	6	5.7	
**Mode of delivery**				<0.001
Vaginal	5,134	44	8.6	
Planned cesarean section	2,836	23	8.1	
Emergency cesarean section	2,254	76	33.7	
**Multiplicity**				<0.001
Single	9,692	113	11.7	
Twin	446	25	56.1	
Triplet	40	2	50.0	
Quadruplet	5	1	200.0	
**Birthweight (gm)**				<0.001
<1,500	259	80	308.9	
1,500–2,499	980	32	32.7	
≥2,500	8,987	32	3.6	
**Gestational age**				<0.001
Full-term	9,164	30	3.3	
Pre-term	1,062	114	107.3	
**Gender of baby**				0.217
Female	4,740	59	12.4	
Male	5,482	84	15.3	

### Newborns' Characteristics

A total of 10,328 (10,226 LB and 102 stillbirths) were registered in the five hospitals (68.4% from the three MOH hospitals, 14% from the teaching hospital, and 18% from the private hospital). Of all births, 46% were females. Almost 95% of total births were singleton, 87% weighed 2,500 grams or more, 10% weighed 1,500–2,499 grams, and 3% weighed <1,500 grams.

### Neonatal Mortality Rate (NMR)

**Figure 1:**
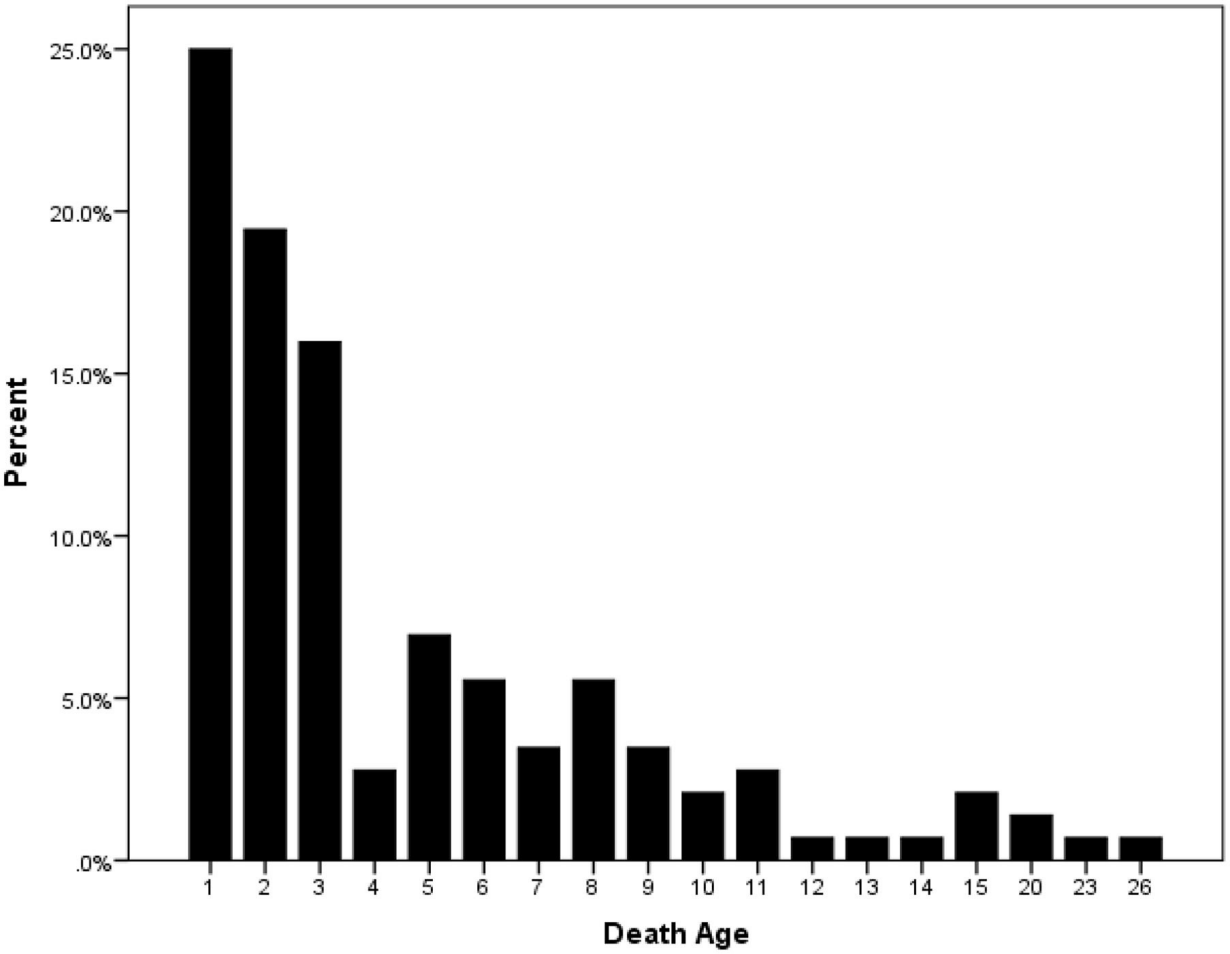
Neonatal death percentages according to age in days in Jordan.

Of the total 10,226 LB, 144 were neonatal deaths. The overall NMR rate was 14.1 per 1,000 LB. Of neonatal deaths, 76% were early neonatal deaths and 24% were late neonatal deaths. Almost 25% of neonatal deaths occurred in the first day, 19% in the second day, 16% in the third day, 3% in the fourth day, 7% in the fifth day (Figure 1).

Table 1 shows the NMR according to the maternal, clinical and relevant characteristics. The NMR per 1,000 LB varied significantly according to mother's educational level, multiplicity, birthweight, mode of delivery, and gestational age. However, the neonatal deaths did not vary significantly according to health sector and mother's age, income, and working status.

### Risk Factors of NMR

Multivariable analysis (Table 2) showed that the odds of deaths in the Ministry of Health hospitals were almost 21 times (OR = 20.8, 95% CI: 2.8, 153.1) higher than that in private hospitals. The odds of death among very low birthweight (<1,500) were 31.8 (95% CI 18.8, 53.8) higher than those with birthweight ≥2,500 grams. Also, pre-term infants were significantly more likely (OR = 13.0; 95% CI: 7.8, 21.6) to die during the neonatal period compared to full-term babies. The odds of neonatal mortality was significantly higher among babies born to housewives compared to those who were born to employed women (OR = 2.7; 95% CI: 1.2, 6.0).

**Table 2:** Multivariable analysis of factors associated with neonatal mortality.

	**OR**	**95% Confidence interval**		***p*-value**
**Birthweight**				
<1,500	31.8	18.8	53.8	<0.0001
1,500–2,499	2.4	1.4	4.3	<0.002
≥2,500	1.0			
**Gestational age**				
Pre-term	13.0	7.8	21.6	<0.001
Full-term	1.0			
**Delivery hospital**				
Private	1.0			
Teaching	4.1	0.5	32.1	0.179
Ministry of Health	20.8	2.8	153.1	<0.003
**Mother occupation**				
Housewife	2.7	1.2	6.0	0.019
Employed	1.0			

### Causes of Neonatal Mortality

Table 3 shows the main causes of neonatal deaths in Jordan. The main leading cause of death was respiratory and cardiovascular disorders which contributed to 43% of pre-discharged deaths and 33% of post-discharged deaths. Of all neonatal deaths classified as respiratory and cardiovascular diseases, the majority (45/55; 81.8%) were due to respiratory distress of newborn, 8 (14.5%) were due to pulmonary hemorrhage originating in the perinatal period, and 2 (3.6%) were due to neonatal aspiration syndromes. The second leading cause of death was low birthweight and pre-term which contributed to 33% of pre-discharged deaths and 42% of post-discharged deaths, followed by congenital malformation deformations and chromosomal abnormalities which contributed to 19% of pre-discharged deaths and 8% of post-discharged deaths. Congenital malformations of heart were the most congenital malformations (7/24, 29.2%)

**Table 3:** Main causes of pre-discharged and post-discharged neonatal mortality.

**Fetal causes**	**Total**	**Pre-discharged *N* (%)**	**Post-discharged *N* (%)**
N1-Congenital malformations and chromosomal abnormalities	24	23 (19%)	1 (8%)
- Q01-Encephalocele	1		
- Q03-Congenital hydrocephalus	2		
- Q20 or Q22 or Q23 or Q24- Congenital malformations of cardiac chambers and connections, pulmonary and tricuspid valves, and other congenital malformations of heart	7		
- Q30-Congenital malformations of nose	1		
- Q33-Congenital malformations of lung	3		
- Q37-Cleft palate with cleft lip	2		
- Q38-Other congenital malformations of tongue mouth and pharynx	1		
- Q45-Other congenital malformations of digestive system	1		
- Q60, Q63-Renal agenesis and other reduction defects of kidney, Other congenital malformations of kidney	2		
- Q74-Other congenital malformations of limb(s)	1		
- Q86-Congenital malformation syndromes due to known exogenous causes not elsewhere classified	1		
- Q87-Other specified congenital malformation syndromes affecting multiple systems	1		
- Q89-Other congenital malformations not elsewhere classified	1		
N2-Disorders related to fetal growth (P05-Slow fetal growth and fetal malnutrition)	1	0 (0%)	1 (8%)
N3-Birth trauma (P10-Intracranial laceration and hemorrhage due to birth injury)	1	1 (1%)	0 (0%)
N4-Complications of intrapartum events	3	3 (3%)	0 (0%)
- P20-Intrauterine hypoxia	1		
- P21-Birth asphyxia Note: This category is not to be used for low Apgar score without mention of asphyxia or other respiratory problems	2		
N5-Convulsions and disorders of cerebral status (P90-Convulsions of newborn)	1	1 (1%)	0 (0%)
N6-Infection (P23-Congenital pneumonia Incl.: infective pneumonia acquired in utero or during birth)	1	1 (1%)	0 (0%)
N7-Respiratory and cardiovascular disorders	55	51 (43%)	4 (33%)
- P22-Respiratory distress of newborn	45		
- P24-Neonatal aspiration syndromes Incl.: neonatal pneumonia resulting from aspiration	2		
- P26-Pulmonary hemorrhage originating in the perinatal period	8		
N8-Other neonatal conditions		1 (1%)	1 (8%)
- P80-Hypothermia of newborn	1		
- P83-Other conditions of integument specific to fetus and newborn	1		
N9- Low birthweight and pre-term (P07-Disorders related to short gestation and low birth weight not elsewhere classified)	44	39 (33%)	5 (42%)
Total		120 (100%)	12 (100%)
**Maternal causes**			
M1-Complications of placenta cord and membranes (P02-Fetus and newborn affected by complications of placenta cord and membranes)	18	17 (55%)	1 (100%)
M2-Maternal complications of pregnancy (P01-Fetus and newborn affected by maternal complications of pregnancy)	8	8 (26%)	0 (0%)
M3-Other complications of labor and delivery (P03-Fetus and newborn affected by other complications of labor and delivery)	1	1 (3%)	0 (0%)
M4-Maternal medical and surgical conditions	5	5 (16%)	0 (0%)
- P00-Fetus and newborn affected by maternal conditions that may be unrelated to present pregnancy	4		
- P04-Fetus and newborn affected by noxious influences transmitted via placenta or breast-milk Incl.: non-teratogenic effects of substances transmitted via placenta	1		
Total (%)	42	31 (100%)	1 (100%)

For the main maternal diseases or conditions affecting fetus/infant, the most common reported condition was complication of placenta, cord, and membrane which contributed to 55% of the 31 deaths that had maternal causes, followed by maternal complications of pregnancy (Fetus and newborn affected by maternal complications of pregnancy), and lastly maternal medical and surgical conditions (Table 3).

## Discussion

The neonatal death rate in the current study is almost similar (14.9 per 1,000 live births) to the 2016 Jordan Perinatal and Neonatal Mortality study using the same cut-off point of gestational weeks (≥20 weeks) (5) indicating that the rate has flatten since 2015 and has not shown a significant decline. Despite the tremendous efforts such as better quality medical care and availability of more advanced medical equipment, still more work is needed to accomplish the Sustainable Developmental Goal by 2030, particularly in regions with high NMR (1) including Jordan.

Our findings were similar to those in Batieha et al. (5) study in which congenital anomalies was a leading cause of death. Literature revealed that although several congenital anomalies could be avoided, they still are important causes of neonatal deaths (16). Congenital malformation was reported constantly across many classification systems (17), which could be preventable by pre-natal folic acid with multivitamin supplements that is proved to decrease the incidence of congenital abnormalities such as neural tube defects (18, 19).

One of the most powerful predictors of neonatal mortality is gestational age at birth.

There is a significant variation in mortality between babies born at 24 weeks and those born at full term (20), reflecting the great impact of immaturity on newborn survival. An exposure that increases pre-term births will therefore increase neonatal mortality. Other causes of neonatal death are congenital malformations, birth trauma, birth asphyxia, and hospital-acquired infection (21, 22). Some risk factors were identified in a recent national study including pre-maturity, low birth weight, maternal age <20 years, history of neonatal death or stillbirth, pre-eclampsia, scarce antenatal care, congenital anomalies, and gestational age before 37 weeks (5).

Assessing the magnitude and etiology of these important events and predicting their risk factors begin with accurately defining and reporting perinatal deaths (23, 24). A strategy for regionalized and cohesive perinatal network should be developed (10, 11) to reduce perinatal morbidity and mortality and improve survival for pre-term infants and other high-risk newborns. Mortality data should be available by geographical area, rural or urban, place of death, timing, underlying cause, and other data such as socio-economic status (25). This can help stakeholder to detect priorities and plan and monitor progress.

Similar to our findings, previous studies also showed that a strong predictor of neonatal death is immaturity as usually reflected by the age in gestational weeks at birth. Neonatal mortality can differ significantly between pre-mature babies and their counterparts full-term infants born at 39–40 weeks of gestation (20). Moreover, the findings of the national Jordan Perinatal and Neonatal Mortality study (5) are congruent with our findings where pre-maturity, gestational age before 37 weeks, low birth weight, multiple pregnancy were the most common risk factors associated with neonatal deaths. Low birth weight may result from both fetal growth restriction and pre-term birth, which are associated with placental dysfunction and subsequent poor fetal outcomes (25).

Previous literature reported similar findings. For instance, a study conducted in 60 low and middle income countries found that NMR was significantly higher among twins vs. singleton newborn babies even after adjusting for birth weight (26). Another study in Bangladesh also found that NMR was much higher among newborn babies born before 34 gestational weeks, twins or triplets, and first child in the family (27).

Congruent with the current study findings, previous studies showed that emergency CS was associated with higher NMR and that cesarean section rates higher than 10% are not associated with reduction in NMR, and hence should be avoided as much as possible (28). It is worth mentioning that the NMR has increased significantly in the last three decades including Jordan, surpassing the WHO recommendations of 10–15% CS as the maximum rates (29, 30).

Interestingly, the current study showed that NMR did not vary significantly according to mother' age, income, and working status but mother's high school or less of education was associated with higher rates of neonatal deaths probably due to less awareness about how and when to access medical care, especially in emergency situations as well as higher influence by family traditions and culture. Incongruent with our findings, maternal age of 30–35 years was associated with higher NMR (27, 31). The latest national study showed that maternal age <20 years was associated with higher rates of neonatal deaths (5). However, some research suggested that advanced maternal age is associated with placental dysfunction that may increase the risk of neonatal deaths and stillbirths (32) or to existing maternal medical condition (33). Also, newborn babies of richer families who also have a high educational level have higher chances to survive than those born to a poor family with lower educational level (34).

Despite the fact that the majority of neonatal deaths can be prevented with efficient interventions, such as access to emergency obstetric and neonatal care (35), some disadvantaged women and newborns who are most vulnerable to death and chronic morbidity have poor access to vital healthcare services (36, 37). Nonetheless, understanding the social and geographical pattern of NMR is crucial for stakeholders to increase access to effective interventions with focus on the poorest populations (36, 38). This will ensure that every pregnant woman and newborn baby have equal access to lifesaving interventions (39).

Complications of placenta was also found to be associated with higher NMR. Placental dysfunction is linked to intrauterine growth restriction, pre-term birth, and birth defects (40) resulting in inadequate oxygen supply to the fetus and thus increasing the probability of pre-term births and/or low birth weight.

Our findings are somehow congruent with the 2016 national study that revealed maternal diseases such as pre-eclampsia, mother's hospitalization during the current pregnancy, and poor antenatal care can all lead to neonatal deaths. It is surprising that in the national study, only a third of neonatal deaths had received optimum medical care (5). Other studies conducted in low-income countries like Pakistan have also specified several contributing factors to neonatal deaths such as inadequate training, insufficient medical care, low competence of healthcare providers and a lack of resources (41). Nonetheless, the national study showed also that a large proportion of neonatal deaths are preventable or possibly preventable thus providing optimal intrapartum, and direct post-partum care is likely to result in reduction of NMR (5).

However, not all births are registered in Jordan, especially if the birth results in stillbirth or early neonatal death before discharge from the hospital and the majority of neonatal deaths are not reported either (10, 11). About 30% of children <5 years do not have a birth certificate (42), and parents do not usually issue a death certificate for the majority of neonatal deaths (36). In the absence of reliable and standardized vital registration and administrative data in many countries, modeling of neonatal mortality rates remains necessary for public health policy and priority setting and monitoring.

Thus, there is a lack of credible data on causes of stillbirths and neonatal deaths making it challenging to develop appropriate interventions to avoid such deaths. The current study fills the gap in such data and hence, encourage stakeholders and policy makers to design and implement timely, evidence-based interventions to regions that register high number of stillbirths and neonatal deaths.

In the current study, having a neonatal mortality rate of 14.1 per 1,000 total births, which is somehow similar to the latest national study indicates that Jordan still falling behind in achieving the SDG of reducing NMR (5). The current study, therefore, highlights an immediate attention to accelerate appropriate efforts to prevent such deaths. This is vital as recent literature reported that with no improvements in neonatal mortality, 27·8 million neonates will die in the period from 2018 to 2030 (1). Yet, if policy makers initiate and implement interventions and improve quality of care to the point that NMR–in the countries that are still behind- would match the SDG target, then 5 million newborn babies could survive. A particular emphasis need to be toward births because a third of all neonatal deaths occur on the day of birth globally and about three-quarters of neonatal deaths occur during the first week of life (36, 43).

### Limitations of the Data

Despite the several strengths of the study mentioned earlier in the Methods section, the study has some limitations that need to be acknowledged. The JSANDS system did not include all hospitals in Jordan, and this could have limited the generalizability of the findings at a national level. However, the five selected hospitals were from three major governorates in Jordan and cover the vast majority of births and deaths occur in all regions in Jordan. Two of these hospitals are large university teaching, referral hospitals, and two of them are only specialized with maternal and child health.

The data exported from the JSANDS for this study were over a period of 6 months, which could have hindered estimating the accurate NMR in Jordan. However, even with this relatively short period, the JSANDS was able to provide an accurate estimates of the rate of neonatal mortality and related causes of deaths, as documented in previous national literature. We will utilize the data registered in the JSANDS in the future to run periodic, perhaps annual, analysis of the rate, causes, and risk factors of Neonatal mortality rate in Jordan. Analyzing the data over a longer period is expected to provide more reliable rates of the current situation in Jordan. Nonetheless, even with the current limitation of the study, our findings, using an innovative electronic surveillance system, are considered a baseline for stakeholders to start developing appropriate interventions and policies to decrease neonatal mortality in Jordan, especially preventable ones.

## Conclusion

The main aim of the current study was to test the association of neonatal and maternal risk factors contributing to neonatal deaths, some of which are preventable, need to be incorporated in policies aimed at reducing NMR. Based on the current findings, the majority of neonatal deaths occurred in the current study could have been prevented with regular antenatal visits, in which all possible diseases or complications of pregnant women or fetal anomalies need to be fully documented and monitored with appropriate and timely medical intervention to minimize such deaths. Specialized care provided to low birthweight neonates and those with respiratory problems by experienced healthcare providers is vital. Additionally, the current study reported the findings of NMR extracted from a national neonatal and stillbirth surveillance system. As the majority of neonatal deaths are not reported in Jordan, investing in the health information systems to improve data registration will encourage appropriate use of interventions to reduce NMR. Finally, there is a need to design and implement evidence-based interventions to mothers and newborn babies who most need it.

## Data Availability Statement

The raw data supporting the conclusions of this article will be made available by the authors, without undue reservation.

## Ethics Statement

The studies involving human participants were reviewed and approved by The study was ethically approved by the Institutional Review Board (IRB) at Jordan University of Science and Technology and the Ministry of Health in Jordan. The data used in this manuscript was retrieved electronically from the JSANDS surveillance system (www.jsands.jo). Written informed consent for participation was not required for this study in accordance with the national legislation and the institutional requirements.

## Author Contributions

NA-S: data collection, writing, original draft preparation, reviewing, and editing. YK: principal investigator, conceptualization, methodology, project administration, and funding acquisition. KS, MA, and AB: data collection, writing, reviewing, and editing. All authors have approved the final version of the manuscript.

## Conflict of Interest

The authors declare that the research was conducted in the absence of any commercial or financial relationships that could be construed as a potential conflict of interest.
